# Supplementary material for: PAR1 Scaffolds TGFβRII to Downregulate TGF-β Signaling and Activate ESC Differentiation to Endothelial Cells
Source: Stem Cell Reports. 2016 Nov 17;7(6):1050–8. doi: 10.1016/j.stemcr.2016.10.006 (PMC5161529; doi:10.1016/j.stemcr.2016.10.006)
Supplement: Document S1. Figures S1 and S2 [file mmc1.pdf]

Stem Cell Reports, Volume 7

Supplemental Information

PAR1 Scaffolds TGFβRII to Downregulate TGF-β Signaling and Activate ESC Differentiation to Endothelial Cells

Haixia Gong, Shejuan An, Antonia Sassmann, Menglin Liu, Victoria Mastej, Manish Mittal, Wei Zhang, Zhigang Hong, Stefan Offermanns, Jalees Rehman, and Asrar B. Malik

Figure S1. Related to Figure 1

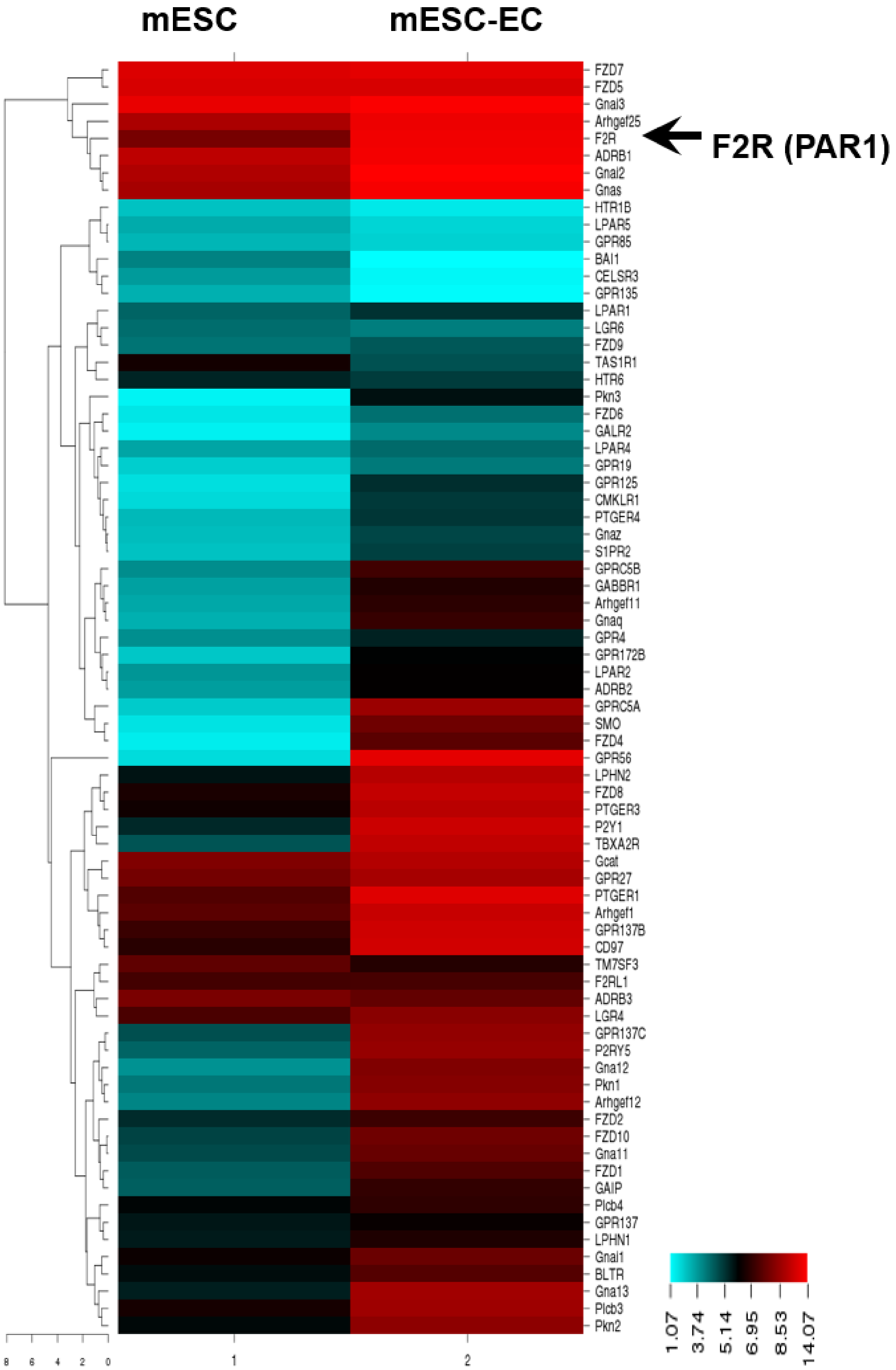

Figure S2. Related to Figure 2

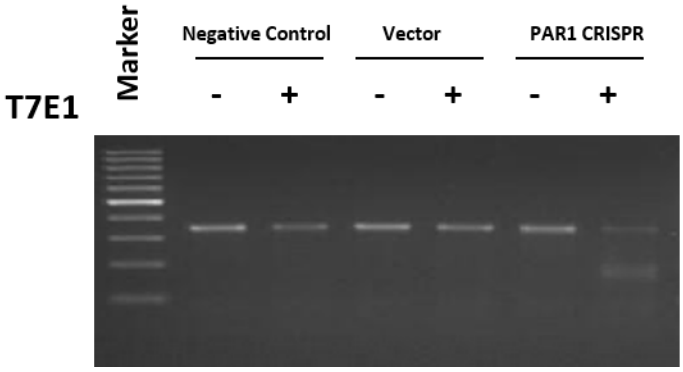

Supplemental figure legends:

Figure S1. Related to Figure 1

Clustering analysis and heat map of 74 GPCR and G protein genes that having greater than 4 copies in undifferentiated mESCs and their corresponding levels in differentiated mESC-ECs. PAR1 (also known as F2R) is highlighted with an arrow.

Figure S2. Related to Figure 2

T7 endonuclease I (T7E1) assay in mESCs demonstrates that T7E1-mediated mismatch cleavage was detected in PAR1-CRISPR mESCs and confirms CRISPR activity.
