# Supplementary material for: PAR1 Scaffolds TGFβRII to Downregulate TGF-β Signaling and Activate ESC Differentiation to Endothelial Cells
Source: Stem Cell Reports. 2016 Nov 17;7(6):1050–8. doi: 10.1016/j.stemcr.2016.10.006 (PMC5161529; doi:10.1016/j.stemcr.2016.10.006)
Supplement: Document S2. Article plus Supplemental Information [file mmc4.pdf]

Stem Cell Reports
Article
ISSCR
OPEN ACCESS

PAR1 Scaffolds TGFβRII to Downregulate TGF-β Signaling and Activate ESC Differentiation to Endothelial Cells

Haixia Gong,[1,2] Shejuan An,[1,2] Antonia Sassmann,[3] Menglin Liu,[1,2] Victoria Mastej,[1,2] Manish Mittal,[1,2] Wei Zhang,[4] Zhigang Hong,[1,2] Stefan Offermanns,[3] Jalees Rehman,[1,2] and Asrar B. Malik[1,2,*]
[1]Department of Pharmacology
[2]The Center for Lung and Vascular Biology
University of Illinois, College of Medicine, 835 South Wolcott Avenue, Room E403, Chicago, IL 60612, USA
[3]Department of Pharmacology, Max-Planck-Institute for Heart and Lung Research, Bad Nauheim 61231, Germany
[4]Feinberg School of Medicine, Northwestern University, Chicago, IL 60611, USA
*Correspondence: abmalik@uic.edu
http://dx.doi.org/10.1016/j.stemcr.2016.10.006

SUMMARY

We studied the function of the G-protein-coupled receptor PAR1 in mediating the differentiation of mouse embryonic stem cells (mESCs) to endothelial cells (ECs) that are capable of inducing neovascularization. We observed that either deletion or activation of PAR1 suppressed mouse embryonic stem cell (mESC) differentiation to ECs and neovascularization in mice. This was mediated by induction of TGFβRII/TGFβRI interaction, forming an active complex, which in turn induced SMAD2 phosphorylation. Inhibition of TGF-β signaling in PAR1-deficient mESCs restored the EC differentiation potential of mESCs. Thus, PAR1 in its inactive unligated state functions as a scaffold for TGFβRII to downregulate TGF-β signaling, and thereby promote ESC transition to functional ECs. The PAR1 scaffold function in ESCs is an essential mechanism for dampening TGF-β signaling and regulating ESC differentiation.

INTRODUCTION

G-protein-coupled receptors (GPCRs), such as PAR1 (Protease Activated Receptor 1, also referred to as CF2R, F2R, TR, and HTR), are transmembrane receptors that transmit extracellular signals into cells by coupling to specific heterotrimeric guanine nucleotide binding proteins (G proteins) and thus mediate an array of responses (Rosenbaum et al., 2009; Vassart and Costagliola, 2011). G-protein-activated pathways constitute the largest class of therapeutic targets (Ding et al., 2015; Thompson et al., 2005). The function ascribed to GPCRs is the result of agonist binding to the receptor, resulting in activation of specific G proteins such as stimulatory $G\alpha s$ and inhibitory $G\alpha i$ subunits, which selectively activate or inactivate effector pathways to mediate the desired responses (Kobilka, 2007; Wess, 1997). However, little is known about the role of GPCRs in mediating the differentiation of stem cells to terminally differentiated cells (Callihan et al., 2011; Kobayashi et al., 2010). To date, work has centered on pathways in adult stem cells such as signals emanating from specialized GPCRs (Frizzled proteins) of the WNT pathway and chemokine receptors such as CXCR4 expressed in stem cells (Holland et al., 2013; Van Camp et al., 2014). The role of GPCR signaling in mediating the differentiation of pluripotent embryonic stem cells (ESCs) into differentiated cells has not been widely explored.

ESCs are critical for regenerative therapies because unlike adult stem cells they expand indefinitely and are ideal for generating mature cells to replace injured tissue. Studies showed that the transcriptional programs underlying ESC differentiation mirror those during embryonic development (James et al., 2005; Shiraki et al., 2014). One example is the differentiation of ESCs into regenerative vascular endothelial cells (ECs), which requires upregulation of the developmental transcription factors such as ER71 (Kohler et al., 2013) and which serves as a window for investigation of signaling pathways mediating vascular regeneration in ischemic tissue. Here we used a GPCR gene expression screen to identify GPCRs expressed in mouse ESCs (mESCs) undergoing differentiation to ECs. We observed that PAR1 was highly upregulated, and further that it was required for EC differentiation. PAR1 functions as a scaffold for the transforming growth factor β (TGF-β) receptor TGFβRII, which thereby dampens SMAD signaling and activates the transition of ESCs to ECs capable of forming new blood vessels.

RESULTS

PAR1 Expression in mESCs Differentiating into ECs

To identify the GPCRs regulating mESC differentiation to ECs, we first performed a GPCR screen made up of 534 GPCRs and related G proteins in mESCs and mESC-derived ECs (mESC-ECs). Three groups of GPCR genes were identified: (1) low expression in undifferentiated mESCs and high expression in mESC-derived ECs; (2) high expression in undifferentiated mESCs and low expression in mESC-ECs; and (3) high expression in both undifferentiated mESCs and mESC-ECs. We focused on the third group consisting of 74 genes having greater than four mRNA copies

This is an open access article under the CC BY license (http://creativecommons.org/licenses/by/4.0/).
CrossMark

A

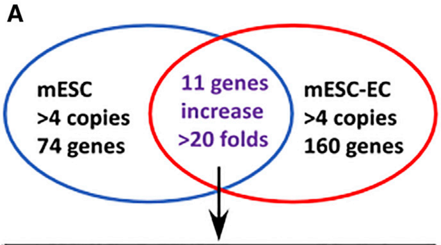

Gene	mESC	mESC-EC	Fold
GPR56	7	2325	352
PAR1	269	12974	48
SMO	6	241	42
GPRC5A	9	378	41
FZD4	6	199	36
Tbxa2r	28	925	33
Gnas	524	16421	31
P2Y1	47	1345	28
Gnai2	711	17238	24
Arhgef12	17	365	21
Gna12	14	293	21

B

Gene	mESC	mESC-EC
PAR1	269	12974
PAR2	166	167
PAR3	2	100
PAR4	0	231

C

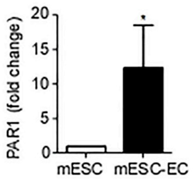

D

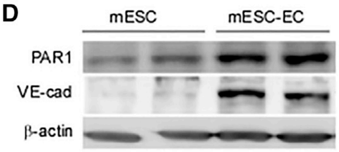

Figure 1. Expression of PAR1 in mESCs and ECs

(A) Gene expression profile of 534 GPCRs and other G proteins showed that 74 and 160 genes in mESCs and mESC-ECs, respectively, expressed greater than four copies of mRNA, within which expression of a subset of 11 listed genes was elevated greater than 20-fold. Results are from two independent experiments.

(B) Comparison of mRNA expression of the four protease-activated receptors (PARs) in mESCs and mESC-ECs.

(C) PAR1 mRNA expression in mESCs and mESC-ECs measured by real-time qPCR shown as mean ± SD (n = 3 independent experiments, $*p < 0.05$).

(D) PAR1 and VE-cadherin protein expression in mESCs and mESC-ECs shown in duplicate samples. VE-cadherin is expressed only in ECs derived from ESCs.

in the undifferentiated mESC state and 160 genes having greater than four copies in mESC-ECs (Figures 1A and S1). Among the highly expressed genes, 11 were upregulated greater than 20-fold in mESC-ECs; specifically the orphan receptor GPR56 (Huang et al., 2008; Jin et al., 2009) was the highest in this category (Figure 1A). PAR1 was the second highest, increasing 48-fold from mESCs to ECs (Figures 1A and 1B). Expression of other PAR genes in ESCs was either unchanged (PAR2) or not evident (both PAR3 and PAR4) (Figure 1B). Furthermore, marked PAR1 upregulation was confirmed by real-time qPCR (Figure 1C) and western blotting (Figure 1D), validating the screen results. VE-cadherin expression was increased in mESC-ECs (Figure 1D), showing the successful transition of ESCs to ECs.

Suppression of PAR1 Expression in mESCs Prevents Differentiation to ECs

To address the role of PAR1 in regulating mESC differentiation to ECs, we knocked down PAR1 using lentivirus-mediated small hairpin RNA (shRNA) inserted into a pLKO1-Puro lentivirus vector (Figure 2A) or deleted PAR1 using CRISPR/Cas9 (Figures 2D and S2). These cells were then differentiated to ECs (Kohler et al., 2013). We observed by fluorescence-activated cell sorting (FACS) analysis that suppression of PAR1 expression markedly reduced the generation of $FLK1^{+}$VE-$cadherin^{+}$ ECs compared with control ESCs (Figures 2B, 2C, and 2E).

To address whether suppression of PAR1 expression per se prevented the generation of ECs, we carried out a rescue experiment in which PAR1 was re-expressed in PAR1-knockdown (PAR1 KD) mESCs by adenovirus transduction using FLAG-tagged human PAR1 (hPAR1). In the rescue experiments, we observed restoration of VE-cadherin and FLK1 expression (Figures 2F–2H), indicating the requisite role for PAR1 in signaling ESC differentiation to ECs. Interestingly, in PAR1 KD studies, we observed that although PAR1 expression was initially suppressed by shRNA it recovered within 4 days (Figure 2F) due to endogenous PAR1 upregulation during EC differentiation (Figure 1C); however, in knockdown cells the initial period of PAR1 KD was in fact sufficient to suppress and delay VE-cadherin and FLK1 expression (Figure 2F).

We also observed that GFP-labeled control ESC-ECs formed functional blood vessels in implanted Matrigel plugs as evident by staining for intraluminal erythrocytes, whereas PAR1-deficient ESC-ECs failed to form any functional vessels (Figure 2I). Importantly, the expression of hPAR1 in mPAR1-deficient ESC-ECs rescued the ability to form vessels (Figure 2I). Quantification of vessels in explanted Matrigel plugs confirmed decreased blood vessel formation in the PAR1-deficient

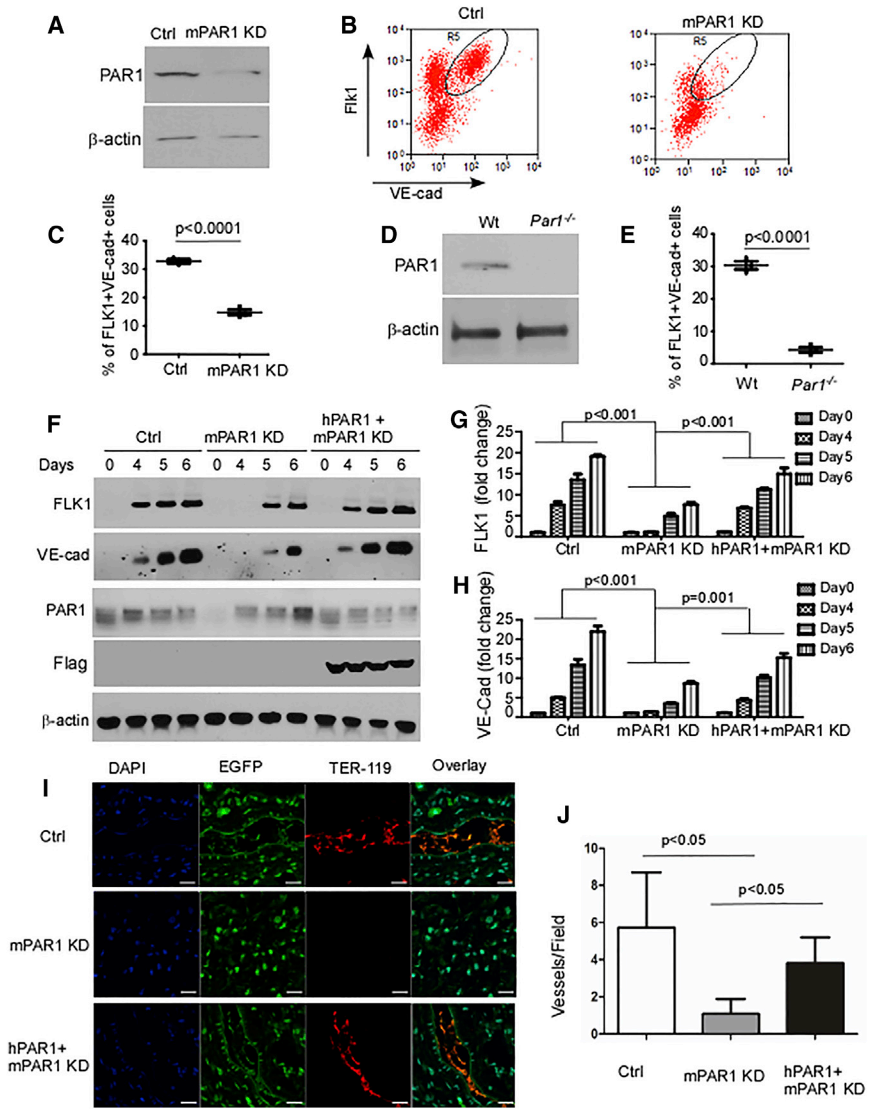

Figure 2. PAR1 Regulates Differentiation of mESCs to ECs

(A) Representative western blots showing expression of PAR1 in scrambled (Ctrl) and PAR1 shRNA lentivirus-treated (KD) mESCs. Results are representative of three independent experiments.

(B) FACS analysis of FLK1 and VE-cadherin (VE-cad) expression in control (Ctrl) or PAR1 KD mESCs at 7 days after applying EC differentiation condition. PAR1 KD suppressed the generation of VE-cadherin$^+$ FLK1$^+$ cells. Mouse IgG1 kappa and rat IgG2a kappa were used as negative controls for VE-cadherin and FLK1 in FACS gating. The outlined cell population indicates double-positive endothelial cells.

(C) Data in (B) presented as percentage of FLK1$^+$VE-cadherin$^+$ ECs (mean ± SD; n = 3 independent experiments).

(D) Representative western blots showing expression of PAR1 in wild-type (Wt) and PAR1-deleted (*Par1*$^{-/-}$) mESCs as induced by CRISPR/Cas9 from three independent experiments.

(E) Quantification of FLK1$^+$VE-cadherin$^+$ ECs by flow cytometry at 7 days after inducing differentiation of Wt and *Par1*$^{-/-}$ mESCs (n = 3 independent experiments).

(F) Transduction of mESCs with control or PAR1 shRNA lentivirus or co-transduction of adenovirus encoding FLAG-hPAR1 followed by differentiation of mESCs into ECs. Cells were harvested at the indicated times and used for western blot analysis. Expression of VE-cadherin, FLK1, and FLAG-PAR1 was determined.

(G and H) Quantification of changes in expression of FLK1 (G) and VE-cadherin (H) proteins (mean ± SD, n = 3 independent experiments).

(I) Representative confocal microscopic images of the erythrocyte marker TER-119 (used to identify functional blood vessels) and EGFP in Matrigel plugs retrieved 7 days after transplantation of purified CD31$^+$ ECs derived from control, PAR1 KD, and hPAR1+PAR1 KD mESCs. Control but not PAR1 KD mESC-ECs formed functional vessels in vivo as indicated by erythrocyte staining, whereas hPAR1 overexpression in PAR1 KD mESCs rescued functional blood vessel formation. Green (EGFP) indicates microvessels generated from transplanted mESC-ECs. Scale bar, 20 μm.

(J) Quantification of vessels seen per field (10×) in H&E staining (n = 3–4 mice per group). The number of vessels decreased in Matrigels from PAR1 KD mESC-ECs and restored in cells re-expressing hPAR1. Data are expressed as mean ± SD of vessels seen (number of vessels was calculated from six fields per Matrigel sample from each mouse; the mean for a group represents the average vessel number from n = 3–4 mice per group).

ESC-ECs and their restoration by re-expression of hPAR1 (Figure 2J).

Inhibition of TGF-β Signaling Overcomes EC Differentiation Block Induced by Deletion of PAR1

We next addressed whether PAR1 enhanced the generation of ECs through inhibition of TGF-β signaling based on the postulated inhibitory role of TGF-β signaling in EC differentiation (Ginsberg et al., 2012; Guo and Chen, 2012; James et al., 2010). We observed that PAR1 KD by shRNA or PAR1 knockout by CRISPR/Cas9 in each case upregulated the expression of TGFβRII (Figures 3A–3C). We also observed that SMAD2 phosphorylation was significantly increased in PAR1 KD ESCs (Figures 3D and 3E), indicating

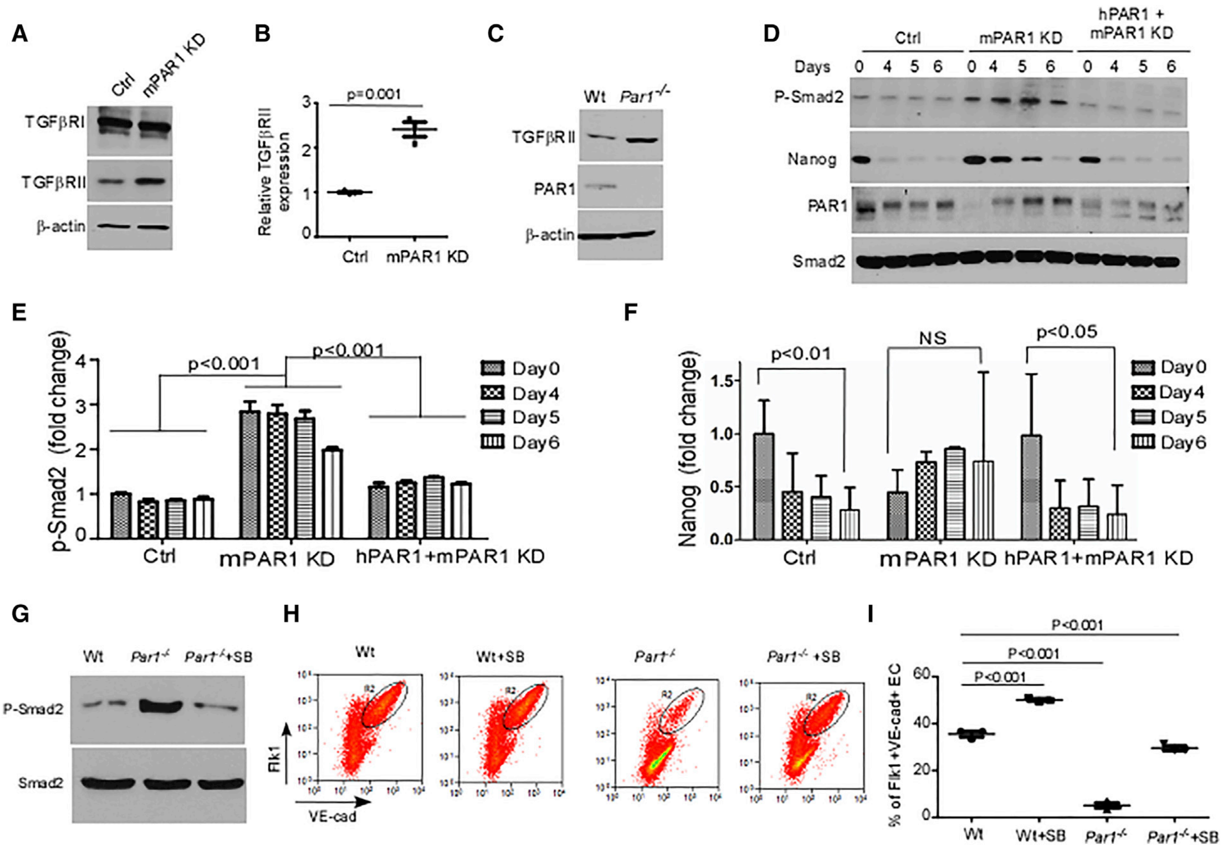

Figure 3. Inhibition of TGF-β Signaling Mediates Transition of mESCs to ECs

(A) Immunoblots of TGFβRI and TGFβRII proteins in mESCs transduced with scrambled control or PAR1 shRNA lentiviruses.

(B) Quantification of TGFβRII expression in (A) showing increased expression in response to PAR1 knockdown (KD) (mean ± SD, n = 3 independent experiments).

(C) Expression of TGFβRII protein in wild-type (Wt) or *Par1*$^{-/-}$ mESCs showing increased TGFβRII expression in PAR1-null cells. Results are representative of two independent experiments.

(D) mESCs transduced with control or PAR1 shRNA lentivirus or co-transduced with adenovirus encoding FLAG-hPAR1, then differentiated into ECs. Expression of NANOG, PAR1, and phospho-SMAD2 as well as total SMAD2 was determined. Upon knockdown of PAR1, expression of NANOG temporally decreased, paralleling the increased phosphorylation of SMAD2.

(E) Quantification of phospho-SMAD2 expression in response to depletion of PAR1 or overexpression of hPAR1 in (D) (mean ± SD, n = 3 independent experiments).

(F) Quantification of NANOG expression in response to depletion of PAR1 or expression of hPAR1 in (D) (mean ± SD, n = 3 independent experiments). NS, not significant.

(G) Augmented phospho-SMAD2 expression as determined by western blot in *Par1*$^{-/-}$ mESCs, which was prevented by treatment with TGFβRI inhibitor SB-431542. Results are representative of two independent experiments.

(H) Representative FACS data showing FLK1^{+}VE-cadherin^{+} ECs derived from Wt and *Par1*$^{-/-}$ mESCs at 7 days after applying EC differentiation conditions in the presence or absence of SB-431542. Mouse IgG1 kappa and rat IgG2a kappa were used as negative controls for FACS gating. The outlined cell population indicates double-positive endothelial cells.

(I) Percentage of FLK1^{+}VE-cadherin^{+} cells derived from FACS experiments in (H) (mean ± SD, n = 3 independent experiments).

the downstream activation of TGF-β signaling. Deletion of PAR1 by CRISPR/Cas9 similarly enhanced SMAD2 phosphorylation (Figure 3G). However, re-expression of hPAR1 reduced SMAD2 phosphorylation to basal levels (Figures 3D and 3E). Importantly, the TGF-β inhibitor SB431542 restored mESC transition to ECs (Figures 3H and 3I), indicating that the effect of PAR1 depletion was secondary to enhanced TGF-β signaling.

As phospho-SMAD2 binds the NANOG promoter and upregulates its expression (Sun et al., 2014), we also determined NANOG expression in PAR1 KD cells. We found that NANOG expression decreased progressively over the 6-day

period in control ESCs undergoing differentiation into ECs (Figure 3F), consistent with the loss of pluripotency. In contrast, NANOG expression remained elevated throughout the differentiation period in PAR1 KD ESCs (Figure 3F), whereas expression of hPAR1 in PAR1 KD ESCs restored the downregulation of NANOG (Figure 3F).

PAR1 Induces EC Generation through Binding of TGFβRII

We next addressed mechanisms of PAR1 regulation of ESC transition to ECs via modulation of TGF-β signaling. Here we first examined the specific effects of PAR1 activation on SMAD2 phosphorylation and the subsequent EC generation. SMAD2 phosphorylation was increased in ESCs following PAR1 activation induced by the PAR1-activating peptide (PAR1-AP) (Figure 4A). Furthermore, this response was prevented by inhibiting TGF-β signaling using SB431542 (Figure 4A). Since PAR1 knockdown or deletion activated TGF-β (SMAD-2 phosphorylation) signaling (Figures 3D, 3E, and 3G) and thereby reduced the generation of ECs (Figures 3H and 3I), we next addressed the effects of PAR1 activation. Here, surprisingly, we observed that PAR1 activation also reduced the generation of VE-cadherin$^+$FLK1$^+$ ECs from ESCs (Figures 4B and 4C).

To address mechanisms by which PAR1 activation downregulated the differentiation of ECs, we examined the interaction of TGFβRII with TGFβRI, a requirement for activation of downstream TGF-β signaling (Huang and Chen, 2012; Zuniga et al., 2005). We observed that the association of TGF-β receptors and subsequent activation of TGF-β signaling was increased in PAR1-deleted mESCs (Figure 4D). In addition, we stimulated mESCs with a specific PAR1-AP (Citron et al., 2016; Gutierrez-Rodriguez and Herranz, 2015) to determine whether activation of PAR1 promoted the binding of TGF-β receptors and thereby activated TGF-β signaling (described in Figure 4A). Here we found that PAR1 activation with PAR1-AP indeed induced TGFβRII/TGFβRI interaction (Figure 4E), an effect also seen in the control experiment following TGF-β1 stimulation (Figure 4E). However, this interaction of TGFβRII with PAR1 was only evident when PAR1 was in the unligated state (Figure 4E). Thus, PAR1 in the inactive state bound TGFβRII and prevented TGFβRII interaction with TGFβRI to inhibit TGF-β signaling.

To validate this model, we studied the effects of overexpressing FLAG-tagged PAR1, and observed that it induced TGFβRII/FLAG-PAR1 association as well as the uncoupling of TGFβRII and TGFβRI (Figure 4F). To identify whether PAR1 can also bind TGFβRI, we expressed FLAG-PAR1 and His-tagged TGFβRI in 293T cells and carried out co-immunoprecipitation experiments using either anti-FLAG-tag or anti-His-tag antibody. However, we failed to detect an interaction between PAR1 and TGFβRI (Figure 4G). Thus, it appears that TGFβRII binds either inactive PAR1 or TGFβRI, but not both simultaneously, and that TGFβRI does not bind PAR1.

Our results support a model in which PAR1 functions as a scaffold for TGFβRII to inhibit downstream TGF-β signaling that is activated by TGFβRII binding to TGFβRI (Figure 4H). The inhibition of TGF-β signaling in turn activates ESC differentiation to ECs and induces neovascularization. Conversely, either depletion of PAR1 or activation of PAR1 results in default dimerization of TGFβRII and TGFβRI to activate TGF-β signaling and hence suppress mESC differentiation to ECs and neovascularization.

DISCUSSION

Studies in *Par1*$^{-/-}$ mouse embryos showed that PAR1 is a key regulator of vascular development; that is, ~50% of *Par1*$^{-/-}$ mice died in utero because of defective vasculogenesis (Griffin et al., 2001). PAR1 utilizes multiple heterotrimeric G proteins, Gαi, Gαq, and Gα12/13, to transmit intracellular signals (Coughlin, 2000; Soh et al., 2010). Only EC-specific *Gα13*$^{-/-}$ embryos died at embryonic days 9.5–11.5 with a phenotype resembling the *Par1*$^{-/-}$ mice (Ruppel et al., 2005). Furthermore, embryos re-expressing Gα13 in ECs did not differ from their *Gα13*$^{-/-}$ littermates and also showed intracranial bleeding (Ruppel et al., 2005), pointing to a key function of PAR1 independent of its associated canonical heterotrimeric G-protein signaling.

In the present study, we carried out an expression profile analysis of GPCRs in mESCs and mESC-derived ECs, and observed inordinately high expression of the orphan receptor GPR56 (Huang et al., 2008) and, importantly, of PAR1 in the ECs generated from ESCs. We focused on PAR1 not only as it is highly expressed in ESCs but also because of its presumptive role in vascular development shown in *Par1*$^{-/-}$ embryos (Griffin et al., 2001). Our results demonstrate that PAR1 expression mediates the differentiation of mESCs to ECs, which were functional as evident by their ability to form vessels in Matrigel plugs in vivo. Intriguingly, downregulation of PAR1 expression as well as direct agonist activation of PAR1 suppressed neovascularization through forcing the association of TGFβRII to TGFβRI, and thereby activating TGF-β signaling (Figure 4H). PAR1 in its inactive state prevented TGF-β signaling by binding TGFβRII, and thus blocked the TGFβRII interaction with TGFβRI required for activation of the TGF-β pathway (Vargel et al., 2016). However, in the absence of PAR1, TGFβRII was free to bind TGFβRI resulting in unfettered TGF-β signaling, which also blocked mESC differentiation to ECs.

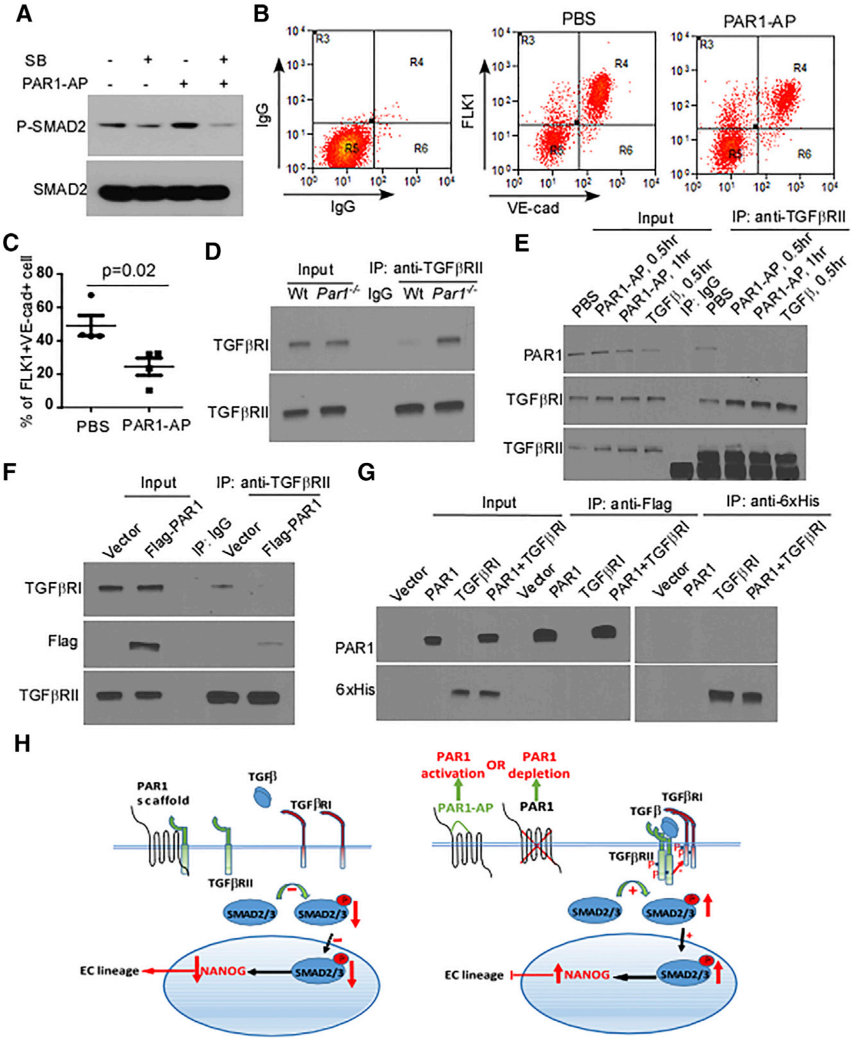

Figure 4. PAR1 Scaffolds TGFβRII to Suppress TGF-β Signaling and Generate VE-Cadherin^{+}FLK1^{+} Cells

(A) Expression of phospho-SMAD2 and total SMAD2 determined by western blot in mESCs challenged with PAR1-AP (15 μM) in the presence or absence of SB-431542 (10 μM). Results are from two independent experiments.

(B) PAR1-induced activation of SMAD2 phosphorylation reduces generation of FLK1^{+} VE-cadherin^{+} cells. FACS analysis of FLK1 and VE-cadherin expression in PBS or PAR1-AP-treated (15 μM) mESCs at 7 days after initiation of EC differentiation protocol. Mouse IgG1 kappa and rat IgG2a kappa were used as negative controls for FACS gating.

(C) Reduced generation of FLK1^{+}VE-cadherin^{+} ECs assessed from data in (B) (mean ± SD, n = 4 independent experiments).

(D) Deletion of PAR1 promotes TGFβRII interaction with TGFβRI. Wild-type (Wt) or *Par1*$^{-/-}$ mESCs were harvested in modified RIPA buffer and cell lysates were immunoprecipitated (IP) with mouse anti-TGFβRII antibody. Immunoprecipitates were blotted with rabbit anti-TGFβRI or anti-TGFβRII antibody. Results are representative of two independent experiments.

(E) Activation of PAR1 with PAR1-AP promotes TGFβRII interaction with TGFβRI. mESCs were stimulated with PBS, PAR1-AP (15 mM), or TGF-β (10 ng/mL), which served as a positive control. Cell lysates were then immunoprecipitated with mouse anti-TGFβRII antibody, and immunoprecipitates were blotted with rabbit anti-PAR1, anti-TGFβRI, or anti-TGFβRII antibody. Results are representative of two independent experiments.

(F) TGFβRII fails to bind TGFβRI in the presence of unligated PAR1. mESCs were transduced with adenovirus encoding empty vector or FLAG-PAR1, and harvested in modified RIPA buffer. Cell lysates were immunoprecipitated with rabbit anti-TGFβRII antibody and immunoprecipitates were blotted with mouse anti-FLAG, anti-TGFβRII antibody, or goat anti-TGFβRI antibody. Results are representative of two independent experiments.

(G) PAR1 in unligated state fails to bind TGFβRI. 293T cells were transfected with plasmids encoding empty vector, FLAG-PAR1, or 6×His-TGFβRI and harvested in modified RIPA buffer. Cell lysates were immunoprecipitated with mouse anti-FLAG or anti 6×His antibody and immunoprecipitates were blotted with rabbit anti-PAR1 or anti-6×His antibody. Results are representative of two independent experiments.

(H) Model describing PAR1 regulation of EC differentiation from ESCs. PAR1 functions as a scaffold, which suppresses TGFβRII activity by competing with TGFβRI for TGFβRII binding. This in turn decreases expression of NANOG and facilitates differentiation toward the EC fate. During PAR1 activation, TGFβRII disassociates from PAR1 and associates with TGFβRI, resulting in TGF-β pathway activation and SMAD2 phosphorylation. In the absence of PAR1, TGFβRII is able to freely associate with TGFβRI to activate TGF-β signaling, and thereby inhibit ESC differentiation to ECs.

In contrast to PAR1, expression of PAR2 (another PAR family member) was not increased in ECs derived from ESCs. PAR3 and PAR4 are also like PAR1 in that they are ligated by thrombin or specific PAR3 and PAR4 agonists (Dery et al., 1998), but they were not significantly expressed in ESCs at baseline. Thus, we focused on the role of PAR1 in regulating mESC differentiation to ECs. Although we cannot rule out the contribution of these PAR family members, they would appear to be less important in regulating the transition of ESCs to ECs based

on the 48-fold increase in PAR1 expression compared with the other PARs.

We observed that although PAR1 expression was initially suppressed by shRNA, it recovered within 4–5 days of initiating differentiation due to marked endogenous PAR1 upregulation occurring during this period. The increase in endogenous PAR1 expression was associated with reduced SMAD2 phosphorylation as evident at 6 days. Importantly, however, knockdown of PAR1 during this initial period of differentiation was in fact sufficient to suppress and delay VE-cadherin and FLK1 expression, suggesting that TGF-β signaling is a critical determinant of EC lineage commitment in this phase.

We determined NANOG expression in PAR1 KD ECSs undergoing differentiation to ECs to assess changes in their pluripotency state. NANOG expression decreased in a time-dependent manner in control ESCs undergoing differentiation, indicating loss of pluripotency. In contrast, expression of NANOG was elevated throughout the differentiation period in PAR1 KD ESCs. This finding is consistent with the evidence that phospho-SMAD2 binding to the NANOG promoter upregulates its expression (Sun et al., 2014).

The finding that inhibition of TGF-β signaling overcame the block in EC differentiation induced by upregulated TGF-β signaling is consistent with the role of suppressed TGF-β signaling as a central mechanism facilitating the generation of ECs from ESCs (James et al., 2010). We showed that the inactive PAR1 functioned as a scaffold for TGFβRII, and restrained the dimerization of TGF-β receptors and, subsequently, downstream SMAD signaling. PAR1 scaffolding thus represents a regulatory mechanism in ESC differentiation to the EC lineage.

EXPERIMENTAL PROCEDURES

Reagents

J1 mESC cell line was purchased from American Type Culture Collection. The anti-VE-cadherin antibody (sc-9989), anti-NANOG (sc-134218), anti-TGFβRI (sc-398, sc-33933), anti-TGFβRII (sc-400, sc-17792), anti-PAR1 (sc-5605), anti-6×His rabbit antibody (sc-803), anti-6×His mouse antibody (sc-8036), and anti-α-actin (sc-32251) antibodies were from Santa Cruz Biotechnology. Anti-VE-cadherin antibody (AF1002), recombinant mouse TGF-β1 (7666-MB-005), and Mouse TER-119 antibody (MAB1125, targets erythrocytes) were from R&D Systems. Alexa Fluor 633 goat anti-rat immunoglobulin G (IgG) (H+L) (A21092, Life Technologies) was used as secondary antibody for detecting TER-119. Collagen IV-coated 6-well plate (354428) and Matrigel were from BD Biosciences. Anti-CD31 antibody (550274) was purchased from BD Biosciences/Pharmingen. Anti-FLK1 antibody (136404) was purchased from Biolegend. Anti-SMAD2/3 (3102), anti-phospho-SMAD2 (3108), and anti-FLK1 (2479) antibodies were from Cell Signaling Technology. The PAR1 agonist peptide (TFLLRNPNDK-NH_2) was synthesized and purified at the Research Resource Center at the University of Illinois, Chicago.

GPCR Screening

mRNA was isolated from mESC and FACS-sorted FLK1$^+$/VE-cadherin$^+$ mESC-ECs. cDNA was generated and a GPCR screen was performed in S.O.'s laboratory. Real-time qPCR was performed using the Universal ProbeLibrary, LightCycler 480 Probes Master, and LightCycler 480 II (Roche Applied Sciences). Genomic DNA from mouse tissue was used for quantification. All primer sequences and probes used in the GPCR screen are listed in the supplemental Excel files (forward primers in Table S1 and reverse primers in Table S2). The Ct/Cp value of 15 ng cDNA was compared with the Ct value of 3 ng genomic DNA to calculate the copy number of genes in this cDNA library.

Cell Culture

mESCs were maintained on mitomycin C-treated mouse embryonic fibroblast (MEF) feeders in mESC growth medium. Before differentiation, mESCs were cultured on MEF-free gelatin-coated 6-well dishes for 2 days in mESC growth medium (pre-conditioning). To start differentiation, we seeded pre-conditioned mESCs in mouse collagen IV-coated 6-well dishes at a density of 3,000 cells/well in serum-free differentiation medium (75% IMDM, 25% Ham's F12 medium) supplemented with N-2, B-27 (without vitamin A), 0.05% BSA, 4.5×10^{-4} M 1-thioglycerol (MTG), 0.5 mM ascorbic acid, 10 ng/mL BMP-4, 50 ng/mL VEGF165, and 10 ng/mL basic fibroblast growth factor (bFGF) as described by us for 7 days (Kohler et al., 2013).

Plasmid Constructs and Lentivirus Preparation

Human PAR1 cDNA plasmids with FLAG tag were purchased from Addgene (Plasmid #53226) and subcloned into pLVX-IRES-puro lentivirus vector (Clontech) or adenovirus vector Adeno-X3 (Clontech). Human TGFβRII cDNA plasmid with HA tag (24801) and human TGFβRI cDNA plasmid with 6×His tag (19161) were purchased from Addgene. The small double-strand hairpin shRNA for PAR1 was designed by Block-iT RNAi Designer (Invitrogen), synthesized by Integrated DNA Technologies (IDT), and inserted into a pLL3.7 lentivirus vector (Addgene, 11795) or pLKO.1-puro lentivirus vector (Addgene, 8453). PAR1 shRNA in a pLKO1 lentiviral vector with puromycin selection was used to obtain a PAR1-KD population. For in vivo studies, we used PAR1 shRNA or scramble control shRNA in a pLL3.7 lentiviral vector expressing EGFP to track the in vivo fate of the cells. The targeting sequence for mouse PAR1 shRNA is 5′-GGTAGGGCAGTCTACTTAA-3′. The guide RNA targeting sequence in mouse PAR1 gene for Cas9-mediated CRISPR knockout used in this study is 5′-GAACACAATCGTG TACACGG-3′. DNA oligos were synthesized by IDT and cloned into pLx-single guide RNA (sgRNA) lentivirus vector (Addgene, 50662) (Wang et al., 2014). Lentivirus was prepared as reported by Gong et al. (2015) and was used to transduce J1 mESCs in the presence of 6 μg/mL polybrene. FLAG-PAR1 adenovirus was produced and amplified in 293A cells. For the Cas9-mediated CRISPR knockout experiment, sgRNA-expression J1 mESCs generated by 10 μg/mL blasticidin selection were infected with Cas9-EGFP adenovirus (Vector Biolabs) at an MOI of 50 twice, and EGFP-positive cells

were sorted by FACS and seeded in 96-well plates. The subpopulation harboring PAR1 deletion by CRISPR/Cas9 was verified by western blot and T7 endonuclease I (T7E1) assay.

Matrigel Plug Assay

The animal experiments were approved by the Animal Care Committee and the Institutional Animal Care and Use Committee (IACUC) of the University of Illinois, Chicago. Experiments were made according to IACUC and NIH guidelines. EGFP-transduced mESC underwent lentiviral knockdown with scramble control shRNA or PAR1-shRNA. A third group consisted of EGFP-transduced mESC in which shRNA-mediated knockdown was rescued by hPAR1 re-expression. These three ESC groups underwent differentiation into ESC-ECs and were purified by CD31-conjugated magnetic beads. ESC-ECs (5×10^5) were injected subcutaneously into 3-month-old athymic nude mice (Harlan Laboratory) in a suspension of 250 μL of Matrigel supplemented with 50 ng/mL VEGF and 20 ng/mL bFGF. Matrigel plugs were retrieved 7 days after transplantation, equilibrated in 30% sucrose overnight, and embedded in OCT compound before freezing and cryosectioning. For quantification of blood vessels in Matrigel, H&E staining was performed.

Immunofluorescence and Confocal Microscopy

Frozen sections were fixed with 4% paraformaldehyde and permeabilized with 0.1% Triton X-100. Slides were probed with primary antibodies and fluorescence-conjugated secondary antibodies (Alexa Fluor 633, Life Technologies). Images were taken with a Carl Zeiss confocal microscope.

Co-immunoprecipitation Assay

This assay was performed as reported by Gong et al. (2010, 2015). Clarified cell lysates from PAR1-depleted mESCs and control cells or 15 μM PAR1-AP or 10 ng/mL TGF-β1 stimulated cells were incubated with anti-TGFβRII antibody, and subsequently with protein A/G-conjugated Sepharose beads. Co-immunoprecipitated proteins were analyzed by western blot as indicated in figures. In some experiments involved in PAR1, TGFβRI, or TGFβRII overexpression, adenovirus encoding FLAG-PAR1 was used to transduce mESCs, and plasmids encoding FLAG-PAR1, HA-TGFβRII, and 6×His-TGFβRI were used to transfect 293T cells.

Flow Cytometry

This assay was performed on a BD LSRFortessa cell analyzer. For labeling of cell surface proteins, 0.05% trypsin-EDTA detached mESC-ECs were resuspended in 1 mL of differentiation medium and incubated at 37°C for 1 hr. Antibodies diluted in washing buffer (0.2% BSA in PBS) were added afterward and incubated for 1 hr at room temperature. After two washes, the cells were resuspended in washing buffer and analyzed immediately by flow cytometry. Mouse IgG1 kappa and rat IgG2a kappa were used as negative controls for FACS gating.

Statistics

Western blot bands were scanned and analyzed for uncalibrated optical density using NIH ImageJ software. ANOVA and Student's t test (two-tailed) were used to determine statistical significance with a p-value threshold set at <0.05.

SUPPLEMENTAL INFORMATION

Supplemental Information includes two figures and three tables and can be found with this article online at http://dx.doi.org/10.1016/j.stemcr.2016.10.006.

AUTHOR CONTRIBUTIONS

H.G. and A.B.M. designed the experiments. H.G., J.R., and A.B.M. wrote the paper. H.G., S.A., A.S., M.L., V.M., M.M., Z.H., W.Z., S.O., and J.R. performed the experiments and analyzed the data.

ACKNOWLEDGMENTS

The work was supported by NIH grants P01HL60678, R01HL45638, R01HL118068, R01HL90152, R01GM094220, and T32HL007829, and AHA grant 13SDG16910050.

Received: January 5, 2016
Revised: October 14, 2016
Accepted: October 17, 2016
Published: November 17, 2016

REFERENCES

Callihan, P., Mumaw, J., Machacek, D.W., Stice, S.L., and Hooks, S.B. (2011). Regulation of stem cell pluripotency and differentiation by G protein coupled receptors. Pharmacol. Ther. *129*, 290–306.

Citron, B.A., Ameenuddin, S., Uchida, K., Suo, W.Z., SantaCruz, K., and Festoff, B.W. (2016). Membrane lipid peroxidation in neurodegeneration: role of thrombin and proteinase-activated receptor-1. Brain Res. *1643*, 10–17.

Coughlin, S.R. (2000). Thrombin signalling and protease-activated receptors. Nature *407*, 258–264.

Dery, O., Corvera, C.U., Steinhoff, M., and Bunnett, N.W. (1998). Proteinase-activated receptors: novel mechanisms of signaling by serine proteases. Am. J. Physiol. *274*, C1429–C1452.

Ding, H.H., Ni, W.J., Tang, L.Q., and Wei, W. (2015). G protein-coupled receptors: potential therapeutic targets for diabetic nephropathy. J. Recept Signal. Transduct Res., 1–11.

Ginsberg, M., James, D., Ding, B.S., Nolan, D., Geng, F., Butler, J.M., Schachterle, W., Pulijaal, V.R., Mathew, S., Chasen, S.T., et al. (2012). Efficient direct reprogramming of mature amniotic cells into endothelial cells by ETS factors and TGFbeta suppression. Cell *151*, 559–575.

Gong, H., Shen, B., Flevaris, P., Chow, C., Lam, S.C., Voyno-Yasenetskaya, T.A., Kozasa, T., and Du, X. (2010). G protein subunit Galpha13 binds to integrin alphaIIbbeta3 and mediates integrin "outside-in" signaling. Science *327*, 340–343.

Gong, H., Rehman, J., Tang, H., Wary, K., Mittal, M., Chaturvedi, P., Zhao, Y.Y., Komarova, Y.A., Vogel, S.M., and Malik, A.B. (2015). HIF2alpha signaling inhibits adherens junctional disruption in acute lung injury. J. Clin. Invest. *125*, 652–664.

Griffin, C.T., Srinivasan, Y., Zheng, Y.W., Huang, W., and Coughlin, S.R. (2001). A role for thrombin receptor signaling in endothelial cells during embryonic development. Science *293*, 1666–1670.

Guo, X., and Chen, S.Y. (2012). Transforming growth factor-beta and smooth muscle differentiation. World J. Biol. Chem. *3*, 41–52.

Gutierrez-Rodriguez, M., and Herranz, R. (2015). From multiple PAR1 receptor/protein interactions to their multiple therapeutic implications. Curr. Top Med. Chem. *15*, 2080–2114.

Holland, J.D., Klaus, A., Garratt, A.N., and Birchmeier, W. (2013). Wnt signaling in stem and cancer stem cells. Curr. Opin. Cell Biol. *25*, 254–264.

Huang, F., and Chen, Y.G. (2012). Regulation of TGF-beta receptor activity. Cell Biosci. *2*, 9.

Huang, Y., Fan, J., Yang, J., and Zhu, G.Z. (2008). Characterization of GPR56 protein and its suppressed expression in human pancreatic cancer cells. Mol. Cell Biochem. *308*, 133–139.

James, D., Levine, A.J., Besser, D., and Hemmati-Brivanlou, A. (2005). TGFbeta/activin/nodal signaling is necessary for the maintenance of pluripotency in human embryonic stem cells. Development *132*, 1273–1282.

James, D., Nam, H.S., Seandel, M., Nolan, D., Janovitz, T., Tomishima, M., Studer, L., Lee, G., Lyden, D., Benezra, R., et al. (2010). Expansion and maintenance of human embryonic stem cell-derived endothelial cells by TGFbeta inhibition is Id1 dependent. Nat. Biotechnol. *28*, 161–166.

Jin, Z., Luo, R., and Piao, X. (2009). GPR56 and its related diseases. Prog. Mol. Biol. Transl Sci. *89*, 1–13.

Kobayashi, N.R., Hawes, S.M., Crook, J.M., and Pebay, A. (2010). G-protein coupled receptors in stem cell self-renewal and differentiation. Stem Cell Rev. *6*, 351–366.

Kobilka, B.K. (2007). G protein coupled receptor structure and activation. Biochim. Biophys. Acta *1768*, 794–807.

Kohler, E.E., Wary, K.K., Li, F., Chatterjee, I., Urao, N., Toth, P.T., Ushio-Fukai, M., Rehman, J., Park, C., and Malik, A.B. (2013). Flk1+ and VE-cadherin+ endothelial cells derived from iPSCs recapitulates vascular development during differentiation and display similar angiogenic potential as ESC-derived cells. PLoS One *8*, e85549.

Rosenbaum, D.M., Rasmussen, S.G., and Kobilka, B.K. (2009). The structure and function of G- protein-coupled receptors. Nature *459*, 356–363.

Ruppel, K.M., Willison, D., Kataoka, H., Wang, A., Zheng, Y.W., Cornelissen, I., Yin, L., Xu, S.M., and Coughlin, S.R. (2005). Essential role for Galpha13 in endothelial cells during embryonic development. Proc. Natl. Acad. Sci. USA *102*, 8281–8286.

Shiraki, N., Ogaki, S., and Kume, S. (2014). Profiling of embryonic stem cell differentiation. Rev. Diabet Stud. *11*, 102–114.

Soh, U.J., Dores, M.R., Chen, B., and Trejo, J. (2010). Signal transduction by protease-activated receptors. Br. J. Pharmacol. *160*, 191–203.

Sun, L.T., Yamaguchi, S., Hirano, K., Ichisaka, T., Kuroda, T., and Tada, T. (2014). Nanog co-regulated by Nodal/Smad2 and Oct4 is required for pluripotency in developing mouse epiblast. Dev. Biol. *392*, 182–192.

Thompson, M.D., Burnham, W.M., and Cole, D.E. (2005). The G protein-coupled receptors: pharmacogenetics and disease. Crit. Rev. Clin. Lab. Sci. *42*, 311–392.

Van Camp, J.K., Beckers, S., Zegers, D., and Van Hul, W. (2014). Wnt signaling and the control of human stem cell fate. Stem Cell Rev. *10*, 207–229.

Vargel, O., Zhang, Y., Kosim, K., Ganter, K., Foehr, S., Mardenborough, Y., Shvartsman, M., Enright, A.J., Krijgsveld, J., and Lancrin, C. (2016). Activation of the TGFbeta pathway impairs endothelial to haematopoietic transition. Sci. Rep. *6*, 21518.

Vassart, G., and Costagliola, S. (2011). G protein-coupled receptors: mutations and endocrine diseases. Nat. Rev. Endocrinol. *7*, 362–372.

Wang, T., Wei, J.J., Sabatini, D.M., and Lander, E.S. (2014). Genetic screens in human cells using the CRISPR-Cas9 system. Science *343*, 80–84.

Wess, J. (1997). G-protein-coupled receptors: molecular mechanisms involved in receptor activation and selectivity of G-protein recognition. FASEB J. *11*, 346–354.

Zuniga, J.E., Groppe, J.C., Cui, Y., Hinck, C.S., Contreras-Shannon, V., Pakhomova, O.N., Yang, J., Tang, Y., Mendoza, V., Lopez-Casillas, F., et al. (2005). Assembly of TbetaRI: TbetaRII: TGFbeta ternary complex in vitro with receptor extracellular domains is cooperative and isoform-dependent. J. Mol. Biol. *354*, 1052–1068.

Stem Cell Reports, Volume 7

Supplemental Information

PAR1 Scaffolds TGFβRII to Downregulate TGF-β Signaling and Activate ESC Differentiation to Endothelial Cells

Haixia Gong, Shejuan An, Antonia Sassmann, Menglin Liu, Victoria Mastej, Manish Mittal, Wei Zhang, Zhigang Hong, Stefan Offermanns, Jalees Rehman, and Asrar B. Malik

Figure S1. Related to Figure 1

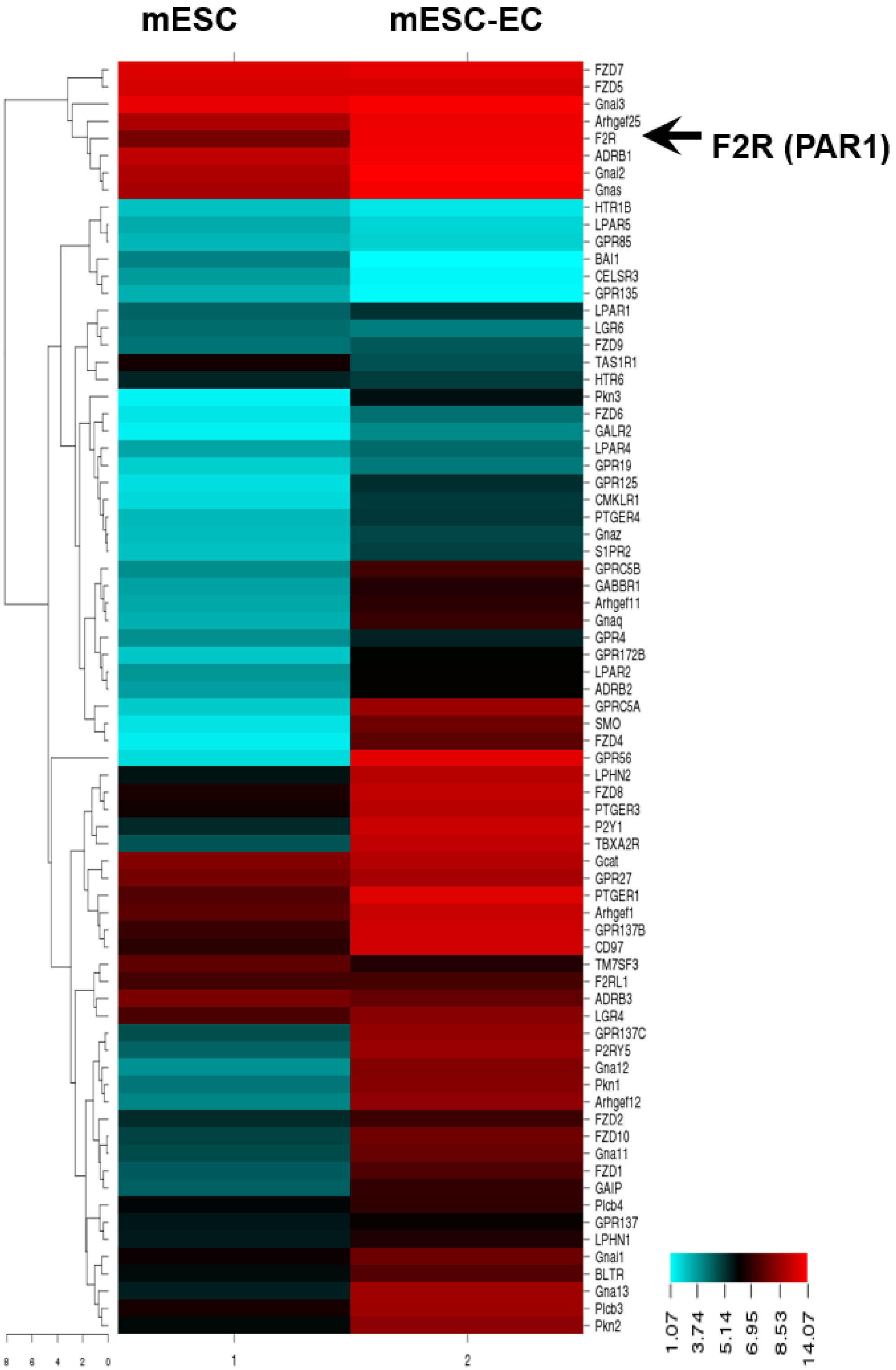

Figure S2. Related to Figure 2

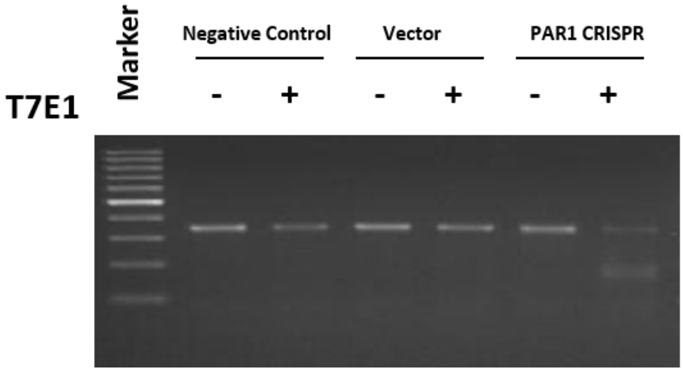

Supplemental figure legends:

Figure S1. Related to Figure 1

Clustering analysis and heat map of 74 GPCR and G protein genes that having greater than 4 copies in undifferentiated mESCs and their corresponding levels in differentiated mESC-ECs. PAR1 (also known as F2R) is highlighted with an arrow.

Figure S2. Related to Figure 2

T7 endonuclease I (T7E1) assay in mESCs demonstrates that T7E1-mediated mismatch cleavage was detected in PAR1-CRISPR mESCs and confirms CRISPR activity.
